# Technical note: A simple method for patient‐specific quality assurance for lateral targets on a 1.5 T MR‐Linac

**DOI:** 10.1002/acm2.14323

**Published:** 2024-03-01

**Authors:** William S. Ferris, Blake R. Smith, Daniel E. Hyer, Joel J. St‐Aubin

**Affiliations:** ^1^ Department of Radiation Oncology University of Iowa Iowa City Iowa USA

**Keywords:** ArcCheck, MR Linac, PSQA, QA platform 1.5T, unity

## Abstract

The Elekta Unity magnetic resonance (MR) linac is limited to longitudinal couch motion and a sagittal‐only laser, which restricts the ability to perform patient‐specific quality assurance (PSQA) intensity‐modulated radiotherapy (IMRT) measurements for very lateral targets. This work introduces a simple method to perform PSQA using the Sun Nuclear ArcCheck‐MR phantom at left and right lateral positions without additional equipment or in‐house construction. The proposed setup places the center of the phantom 1.3 cm vertical and 12.9 cm lateral to isocenter in either the left or right direction. Computed tomography (CT) scans are used to simulate the setup and create a QA plan template in the Monaco treatment planning system (TPS). The workflow is demonstrated for four patients, with an average axial distance from the center of the bore to the planning target volume (PTV) of 12.4 cm. Gamma pass rates were above 94% for all plans using global 3%/2 mm gamma criterion with a 10% threshold. Setup uncertainties are slightly larger for the proposed lateral setup compared to the centered setup on the Elekta platform (∼1 mm compared to ∼0.5 mm), but acceptable pass rates are achievable without optimizing shifts in the gamma analysis software. In general, adding the left and right lateral positions increases the axial area in the bore encompassed by the cylindrical measurement array by 147%, substantially increasing the flexibility of measurements for offset targets. Based on this work, we propose using the lateral QA setup if the closest distance to the PTV edge from isocenter is larger than the array radius (10.5 cm) or the percent of the PTV encompassed by the diode array would be increased with the lateral setup compared to the centered setup.

## 1 INTRODUCTION

The Unity (Elekta AB, Stockholm, Sweden) is a magnetic resonance (MR) guided linear accelerator (linac) that specializes in adaptive radiotherapy.^1^ The couch only moves in the longitudinal direction, therefore daily treatment is performed with plan adaption rather than couch shifts such as is commonly performed on conventional linacs. Treatments are adapted using either an adapt to position (ATP) or adapt to shape (ATS) treatment mode, which reflects a virtual couch shift and a full replan, respectively.^2^, ^3^, ^4^ The treatment field size on the Unity is 22 cm in the longitudinal direction and 57.4 cm diameter in the axial plane at isocenter, indicating that targets can be treated up to 28.7 cm laterally from the center of the bore.

The limitation of no vertical or lateral couch motion causes challenges for patient‐specific quality assurance (PSQA). In addition, the Unity has only a sagittal laser and therefore relies on platforms to align quality assurance (QA) equipment. For intensity modulated radiotherapy (IMRT) PSQA, the Unity relies on a specialized QA platform provided by Elekta to place a cylindrical diode array phantom at the radiation isocenter, which is 14 cm above the hard couch surface and centered laterally on the couch.^5^ However, this platform has a fixed position on the couch, which poses problems for lateral targets. Custom platforms mimicking the Elekta‐provided platform at various lateral or vertical positions can be made, but these require time and resources to manufacture.

The purpose of this work was to develop and evaluate a workflow to enable the measurement of IMRT PSQA for lateral targets using a process that does not require additional equipment beyond what is provided by Sun Nuclear with the ArcCheck‐MR (Sun Nuclear, Melbourne, FL). Requirements of the method were precise positioning accuracy, good setup reproducibility, and a simple setup in the treatment planning system (TPS) such that PSQA plans can be generated quickly in the clinical workflow.

## 2 METHODS

The ArcCheck‐MR is used for Unity IMRT PSQA in our clinic and is used for the proposed workflow. However, the workflow could apply to other MR‐safe phantoms. The ArcCheck‐MR contains a cylindrical array of 1386 diode detectors with 1 cm spacing and has been validated for use in a 1.5T MR field.^5^ The diameter of the active diode array is 21 cm, and the outer diameter of the phantom is 27 cm.

**FIGURE 1:**
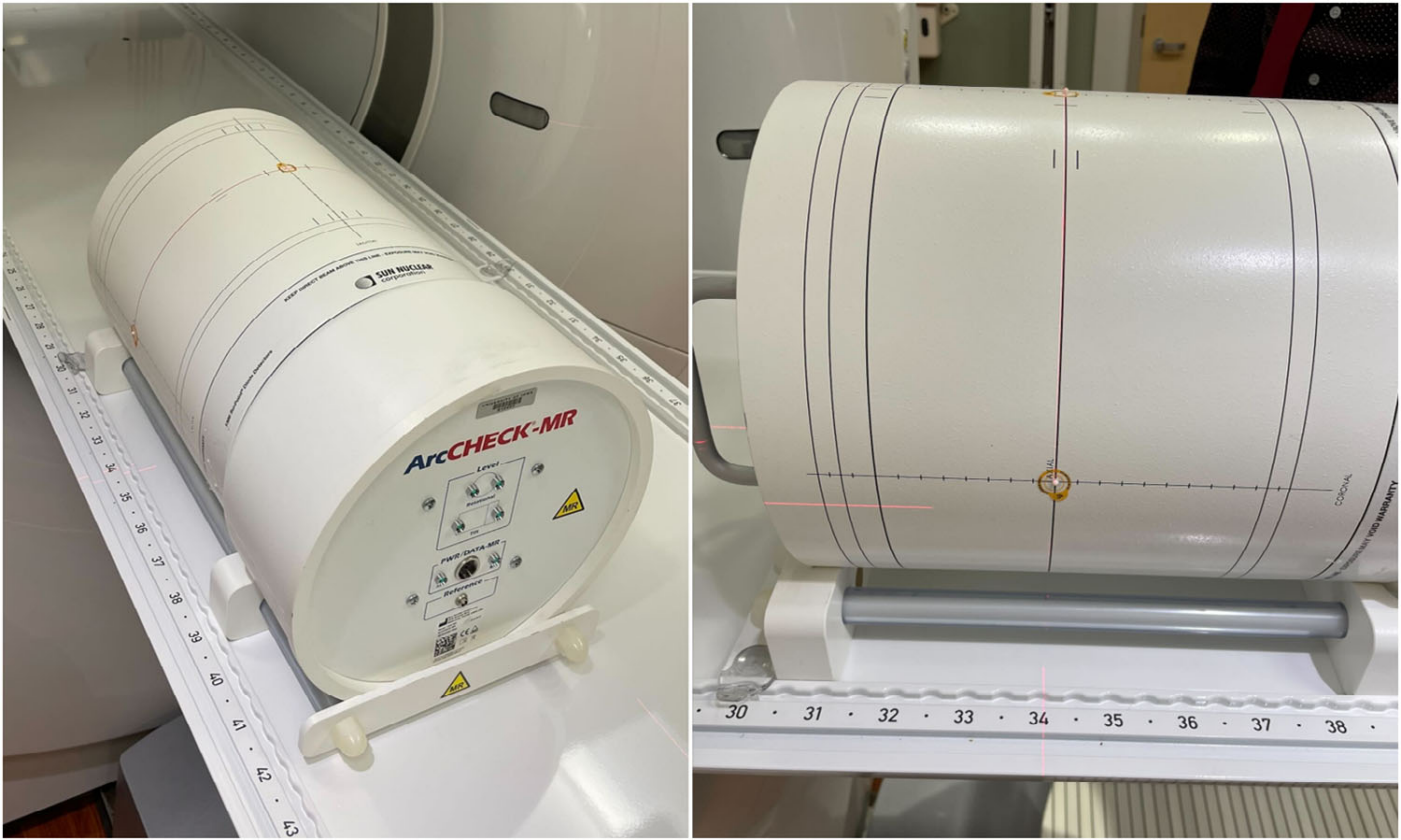
Photographs of the ArcCheck‐MR positioned for a right‐sided IMRT PSQA. The lateral position is set by abutting the holder to the lateral rail, the longitudinal is set by abutting the holder to an index bar, and the vertical position is a constant value of 15.3 cm.

The proposed workflow relies on accurate and reproducible positioning on the Unity treatment table. For our setup, the ArcCheck‐MR is placed on the SNC‐provided, MR‐conditional ArcCheck‐MR holder with the feet removed to create a consistent height above the couch, as shown in Figure 1. This setup places the phantom center 15.3 cm above the couch (1.3 cm above the isocenter). The ArcCheck‐MR and platform are placed abutting the lateral rail of the couch and the inferior edge of an index bar set at index 30, although different index locations could be utilized. The lateral position of the center of the phantom is 12.9 cm from the center of the bore (determined using the TPS). The roll of the phantom is set with the leveling light indicators or by physical measurement of the height of the left and right lateral lines on the phantom above the couch.

The lateral ArcCheck‐MR setup was simulated using the CT scanner to determine the exact offset position. The Unity couch top insert was physically overlaid on the CT couch top and the ArcCheck‐MR lateral setup was simulated as described above. A new QA plan template was created in the TPS using the new CT simulation images. Fiducial ball bearings (BBs) were placed at the crosshair on the left lateral, right lateral, anterior, and posterior locations of the ArcCheck‐MR phantom. These markers were used for alignment and planning in the Monaco TPS (Elekta AB, Stockholm, Sweden). With the index bar at 30, the CT BBs on the center of the phantom were measured to be approximately at index 34, as shown in Figure 1. The CT scans were imported into the QA clinic in the Monaco TPS and the setup and scan reference points were set to the BBs. To maintain consistent calculation conditions as the centered QA plan, the CT image was registered using rigid translations to the centered QA plan and the ArcCheck‐MR external contour was transferred to the lateral datasets. This method ensures the final phantom dataset used for dose calculation is the same dimensions for the lateral plans as for the centered plan. The ArcCheck‐MR phantom body was overridden to an electron density (ED) of 1.192, which is the optimized ED of our phantom in the TPS.^2^ The bars of the platform holding the ArcCheck‐MR were contoured and overridden to an ED of 1.2 based on the average value on the CT.

**FIGURE 2:**
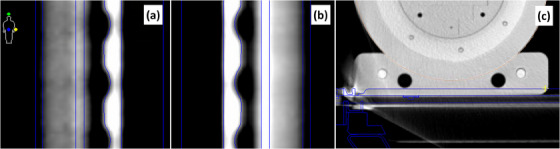
Coronal slices of the right (a) and left (b) rail used for lateral and longitudinal alignment of the couch top relative to the CT in the planning system. Axial slice (c) of the phantom and couch top interface used for lateral and vertical alignment.

The Unity couch was added to the QA plan and aligned with the CT image, shown in Figure 2. The positioning of the couch relative to the CT BBs was used as the final offset of the phantom relative to the center of the bore, which were 1.3 cm vertical and 12.9 cm lateral. The vertical position was placed such that the phantom and hard couch top interface aligned with the contour interface. The longitudinal position was set by entering the CT BB index of 34 and optimizing the “Offset (cm)” parameter upon couch import such that the ridges in the couch contour aligned with the ridges in the couch CT image, shown in Figure 2. A final offset of 3.0 mm was used and was confirmed by physical measurement at CT simulation within 0.5 mm. This 3 mm offset indicates that the CT BBs (a surrogate of the center of the phantom) are 3 mm past index 34. A couch position of 254.0 cm places index 34 at the isocenter plane. Therefore, a couch position of 254.3 cm places the center of the phantom at the isocenter plane.

To assess the reproducibility of the phantom positioning, five repeated CT scans were acquired for both the lateral setup and the centered setup. The phantom and platform were removed from the table and re‐aligned between each image. Image registration was performed in the Velocity AI software (Varian Medical Systems, Palo Alto, CA) between each of the four repeated images and the first image. A registration region of interest (ROI) was placed which included the entire acrylic portion and the superior end of the ArcCheck‐MR. Auto rigid registration including translations and rotations was performed, then the translations were manually tuned using image fusion with the primary as grayscale and the secondary as inverted grayscale.^6^

Clinical plans with lateral targets were retrospectively identified, recalculated on the lateral ArcCheck‐MR QA template, and re‐delivered. The calculation grid was 2 mm, dose was calculated to water, the phantom material lookup table was used, and the Monte Carlo GPUMCD algorithm^7^ with 1% per‐calculation uncertainty was used. The known offset of the phantom from isocenter (1.3 cm vertical and 12.9 cm lateral) was entered into the SNC Patient software (Sun Nuclear, Melbourne, FL) upon importing the dose. This offset is needed to properly assign doses in the DICOM file to the diode array.

## 3 RESULTS

**FIGURE 3:**
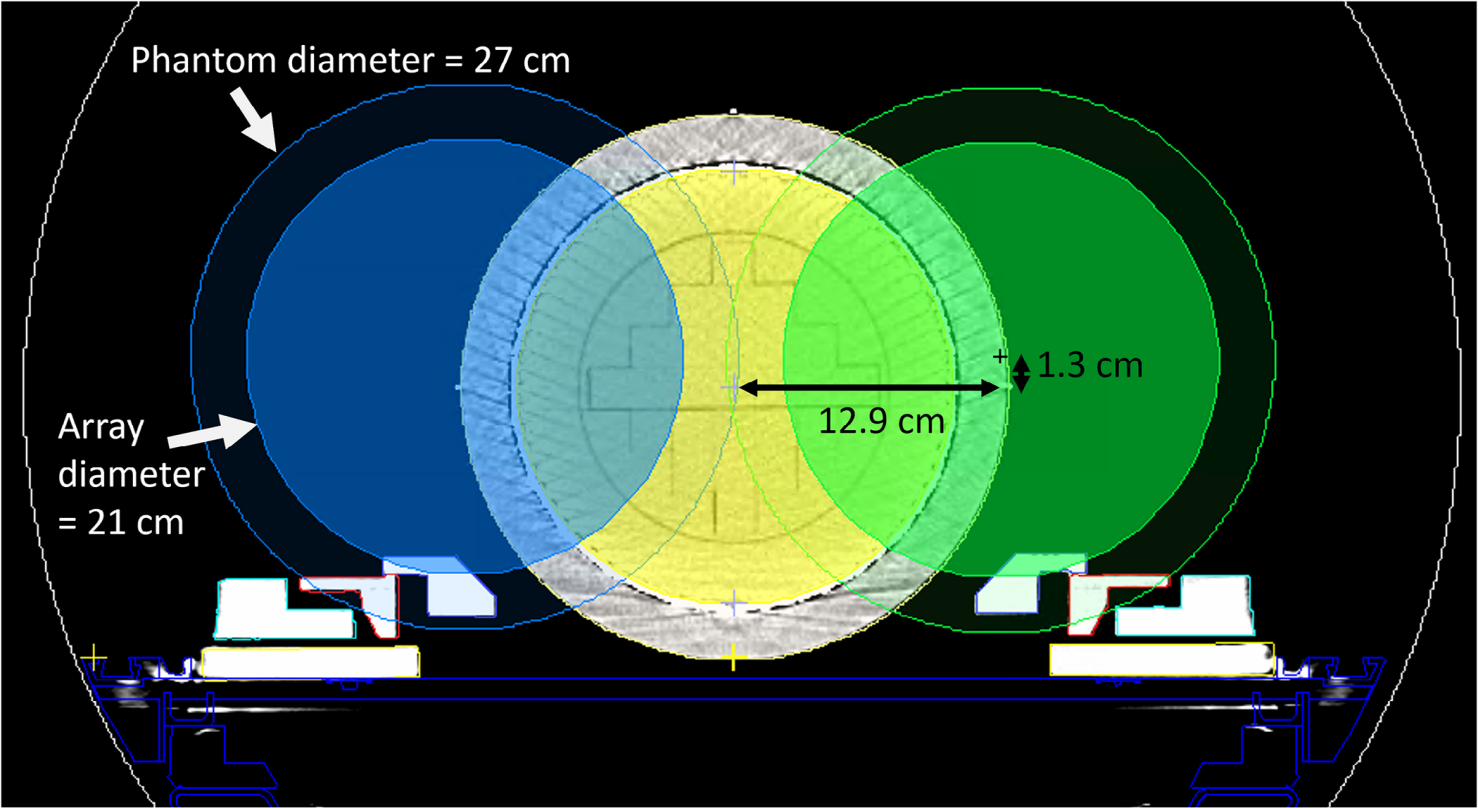
Illustration of the area covered by the three (centered, lateral left & right) QA plans used in our clinic and their location relative to isocenter. The white line indicates the 70 cm bore diameter.

**FIGURE 4:**
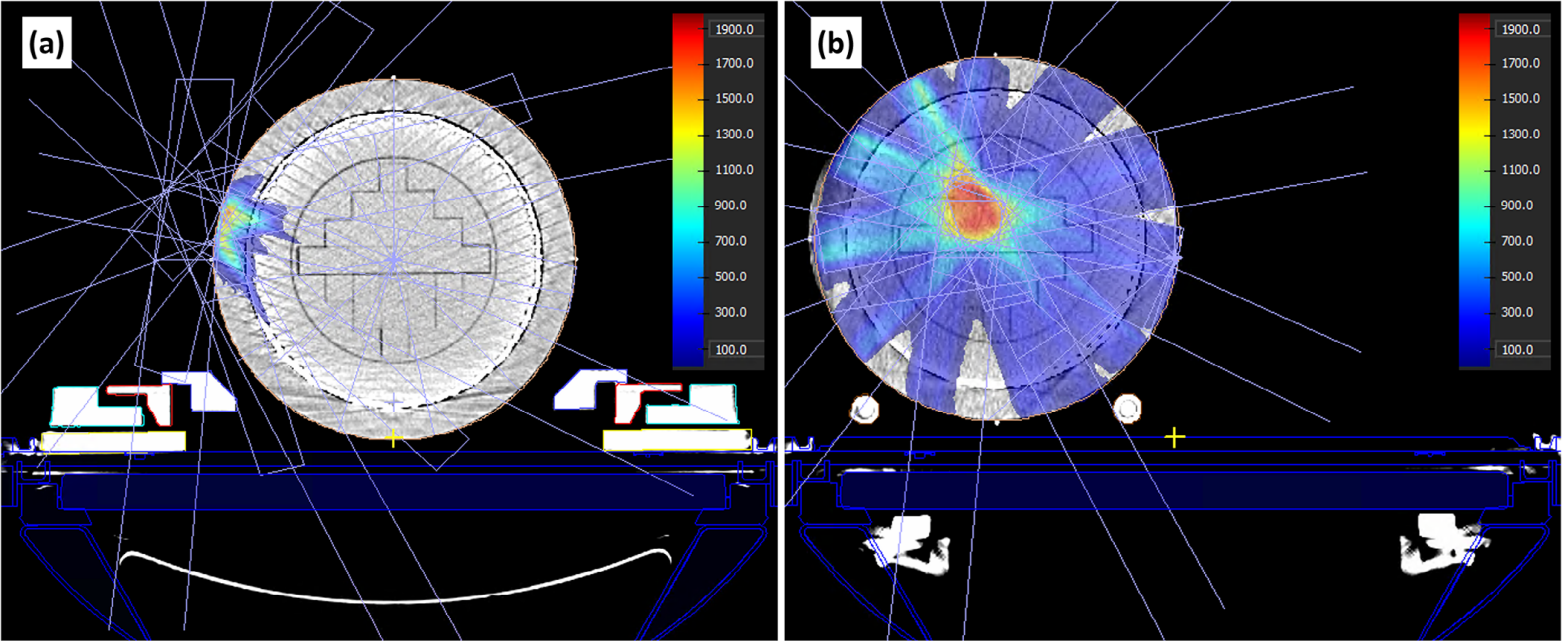
Example PSQA plan for patient 1 calculated on (a) the centered phantom setup, and (b) the right lateral phantom setup.

As shown in Figure 3, adding the left and right lateral QA phantom positions increase the axial area in the bore encompassed by the cylindrical diode array from 346 cm^2^ for the centered plan to 853 cm^2^ total among the three QA positions, a 147% increase. Example PSQA plans on a centered and lateral phantom are shown in Figure 4. The figure shows how there are several beams that entirely miss the diode array for the centered setup, but the beams are within the diode array for the lateral setup.

Statistics for each of the QA plans are shown in Table 1. All gamma pass rates were above the clinical pass criteria of > 90% for 3%/2 mm, and 10% threshold.^8^ The calculate shift function in SNC Patient was not used for the results presented in Table 1. To test the positioning of the phantom, the calculate shift function in SNC Patient was applied to each patient. The pass rates increased for two patients and stayed the same (no better shift found) for two patients. The magnitude of the shift was 1 mm or less. For the four patient plans investigated, the mean lateral PTV position from the center of the bore was −12.1 cm laterally, 1.3 cm vertically, and 12.4 cm vector in the axial plane. The average distance in the axial plane from the center of the bore to the closest edge of the PTV was 10.2 cm. If this distance from the PTV edge to the center of the bore is greater than 10.5 cm (the radius of the diode array), the entire PTV would be outside the diode array using a centered phantom placement, and thus would be a good indicator that a lateral setup should be used.

**TABLE 1:** Summary of the example lateral IMRT QA plans. The PTV centroid and closest edge distances are relative to the center of the bore, with positive directions being patient left and anterior when head‐first supine.

Patient	Centroid LR (cm)	Centroid AP (cm)	Centroid axial (cm)	Closest edge axial (cm)	Volume (cc)	Volume in centered array (%)	Volume in lateral array (%)	Gamma 3%/2 mm
1	−14.55	3.49	14.96	13.16	36.49	0.0	100.0	97.3
2	−12.41	3.73	12.96	11.66	13.14	0.0	100.0	94.6
3	−8.49	−0.29	8.49	6.04	53.73	94.3	100.0	96.4
4	−12.92	−1.86	13.05	9.84	164.37	0.0	100.0	98.6

Another metric that can be used to determine if the lateral measurement would be beneficial is the percentage of the PTV volume that is encompassed in the active diode array. This was computed in the TPS by creating a cylindrical contour with the dimensions of the diode array (21 cm diameter, 21 cm length), and computing the overlap volume with the PTV. The percentage overlap was 100% for the lateral setup for all four patients in this work, and 0% for the centered setup for three of the patients in this work (note that these patients were intentionally selected because of the large lateral shift). For patient 3, the overlap was 100% for the lateral and 94.3% for the centered, indicating that either phantom setup could be used, but the sensitivity of the measurement may be higher with the lateral setup.

The position of the lateral phantom setup was evaluated to be within a range of 0.35, 0.10, and 0.90 mm in the left/right (LR), anterior/posterior (AP), and superior/inferior (SI) directions, respectively. The position of the centered phantom setup on the Elekta platform was found to be reproducible within a range of 0.25, 0.03, and 0.50 mm in the LR, AP, and SI directions, respectively. All rotations were less than 0.15 degrees for both setups.

## 4 DISCUSSION

The systematic and random positional uncertainties of the lateral phantom setup were compared to those of the centered phantom setup on the Elekta‐provided platform. The uncertainty in the positioning of the centered phantom on the Elekta platform, which is positionally indexed to isocenter using MV images and a calibration platform with fiducials,^4^ has been reported to be 0.3 mm. The systematic uncertainty of the lateral QA platform refers to the difference between the TPS‐modeled and actual position of the phantom on the couch. Any difference in these values will result in a dose distribution calculated on a phantom that is in a different position in the TPS than during PSQA delivery. The vertical and longitudinal position of the phantom were verified by physical measurement during CT simulation, which agreed within 0.5 mm with the TPS. The lateral offset was verified by registering the left and right lateral CT images together in Velocity AI. The translation between the images was 25.76 cm, which is within 0.4 mm of twice the lateral offset of 12.90 cm (25.80 cm).

The repeated CT scans indicate that the random setup error of the lateral phantom is slightly larger in the SI direction than the centered phantom on the Elekta platform: 0.9 versus 0.5 mm. This is likely due to the slop in the index bar placement on the couch top. The range of setup uncertainties in the LR and AP directions were similar (0.35 mm or less) for the lateral and centered phantoms. An additional uncertainty to consider is the roll of the phantom, however, this uncertainty is present for all ArcCheck‐MR PSQA measurements. The total uncertainty (systematic plus random) in the position of the lateral phantom is expected to be larger than the centered phantom using the Elekta platform, but the results indicate that positioning is better than 1 mm and that acceptable passing rates can be achieved even without applying shifts in the gamma analysis software.

The method outlined in this work allows for both right and left sided lateral treatments. However, to date, we have not encountered a left sided case requiring a lateral QA measurement. All the cases that have required a lateral measurement have been for liver treatments, thus on the right side. However, it is expected that peripheral IMRT lung treatments would require both left and right lateral PSQA measurements. In addition, we have not yet encountered a case where the PTV would be outside of both the centered and lateral positions (Figure 3). This could potentially occur for a very anterior or posterior target, but we expect this to be rare.

**FIGURE 5:**
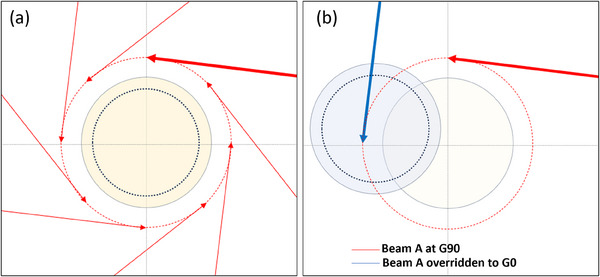
An example of how a lateral phantom increases beam measurability via gantry angle overrides. An off‐axis field from a gantry angle of 90° will (a) miss a centered phantom for all gantry angles, but (b) will intersect a lateral phantom if overridden to gantry angle of 0°.

Another benefit of the lateral QA setup is that it allows for the ability to override gantry angles of the IMRT fields for PSQA purposes to force the beams to intersect the array. This concept is demonstrated in Figure 5. This method is not an option for a centered ArcCheck‐MR setup since the central axis of the phantom aligns with gantry rotation axis. For the lateral ArcCheck‐MR, beams with lateral fields can be overridden to force the field to intersect phantom.

We believe that a lateral PSQA setup is beneficial in scenarios where the distance to the closest edge of the PTV is greater than 10.5 cm, as the centered setup will not encompass the PTV. Patient 3 is an example of a case where either the centered or lateral IMRT PSQA measurement could be used. The distance to the closest edge of the PTV is 6.04 cm, which is less than the array radius of 10.5 cm. The lateral setup covers 100% of the PTV and the centered setup covers 94.3% of the PTV. In this case, it would be better to use the lateral setup since it will increase the sensitivity of the measurement. This type of analysis will be performed for future clinical cases where the position of the PTV is on the edge between the centered and lateral measurement.

The ArcCheck‐MR corrects for the angular dependence of the diodes by calculating the gantry angle with a virtual inclinometer (implied from the raw signal distribution) and applying known dependencies based on the angle between the source and each diode. One disadvantage of placing the phantom laterally is changing the angular correction, since the algorithm assumes the phantom is at isocenter. The maximum *change* in incidence angle between the diode and a ray line is expected for the most anterior diode and gantry angle of zero degrees, which is 5.6 degrees as calculated in Equation (1). Literature indicates that a 5.5‐degree angular error may cause a change in response of 3.5%.^9^ However, 5.6 degrees is the maximum angular error in our setup and therefore the cumulative effect is expected to be much lower for the entire plan. In addition, our results indicate that high gamma pass rates can be obtained despite this effect.

(1) $$\theta =\tan^{-1}\left(\frac{12.9\;\text{cm}}{143.5\;\text{cm}-1.3\;\text{cm}-10.5\;\text{cm}}\right)=5.6^{\circ }$$

## 5 CONCLUSION

We have presented a method to perform PSQA on the Elekta Unity MR‐Linac for cases where the targets are very lateral and would otherwise be difficult to measure using the standard PSQA method with the Elekta provided QA platform. The lateral PSQA setup can be replicated in other clinics provided they have the same phantom and phantom base. We expect the position of our ArcCheck‐MR setup (12.9 cm lateral and 1.3 cm vertical from the center of the bore, and 0.3 cm from index 34 with index bar at index 30) to be very similar to other ArcCheck‐MR and Unity devices, with the only variation being the manufacturing variability of the Unity table and the ArcCheck‐MR holder and the user registration in the TPS. The position of the ArcCheck‐MR should be validated in each clinic before using this method.

## AUTHOR CONTRIBUTIONS

William Ferris and Joel St‐Aubin contributed to the conception and design of the study. William Ferris completed the data collection. All authors contributed to the data analysis. William Ferris and Joel St‐Aubin drafted the and reviewed the manuscript, and Blake Smith and Daniel Hyer reviewed and revised the manuscript. Joel St‐Aubin supervised and managed the project. All authors contributed to the article and approved the submitted version.

## CONFLICT OF INTEREST STATEMENT

Joel St‐Aubin reports honorarium and research funding from Elekta unrelated to this work. Daniel Hyer discloses a consulting relationship with Elekta and research funding from Elekta unrelated to this work. The remaining authors have no conflicts of interest to disclose.
